# Supplementary material for: Causes and consequences of sex-chromosome turnovers in Diptera
Source: Evol Lett. 2026 Mar 12;10(3):253–68. doi: 10.1093/evlett/qrag003 (PMC13231274; doi:10.1093/evlett/qrag003)
Supplement: qrag003_Supplemental_Files [file qrag003_supplemental_files.zip › Supplementary_Datasets.zip!/Supplementary_Datasets/Dataset_2_Graphical_representation_outgroups/Polleniidae_Pollenia_angustigena_chromosomes.pdf]

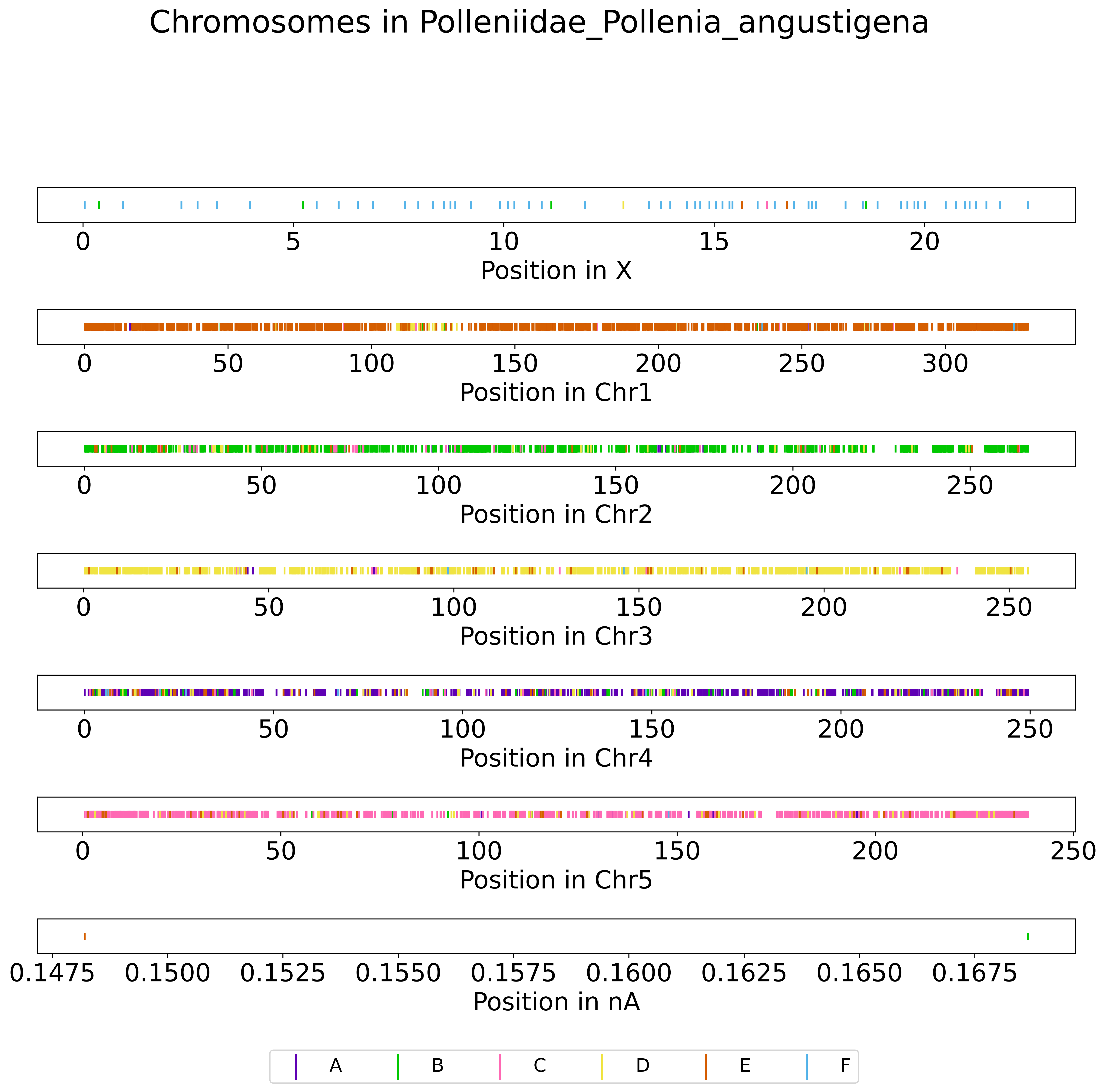

Chromosomes in Polleniidae_Pollenia_angustigena
0 5 10 15 20
Position in X
0 50 100 150 200 250 300
Position in Chr1
0 50 100 150 200 250
Position in Chr2
0 50 100 150 200 250
Position in Chr3
0 50 100 150 200 250
Position in Chr4
0 50 100 150 200 250
Position in Chr5
0.1475 0.1500 0.1525 0.1550 0.1575 0.1600 0.1625 0.1650 0.1675
Position in nA
A B C D E F
